# An eye for an ‘I:’ a critical assessment of artificial intelligence tools in migration and asylum management

**DOI:** 10.1186/s40878-022-00305-0

**Published:** 2022-08-03

**Authors:** Lucia Nalbandian

**Affiliations:** Canada Excellence Research Chair in Migration and Integration Program at Toronto Metropolitan University (formerly Ryerson University), Toronto, Canada

**Keywords:** Migration, Asylum, Artificial intelligence, Efficiency, Vulnerability, Digital technology, United States, United Nations, New Zealand

## Abstract

The promise of artificial intelligence has been originally to put technology at the service of people utilizing powerful information processors and ‘smart’ algorithms to quickly perform time-consuming data analysis. It soon though became apparent that the capacity of artificial intelligence to scrape and analyze big data would be particularly useful in surveillance policies. In the wider areas of migration and asylum management, increasingly sophisticated artificial intelligence tools have been used to register and manage vulnerable populations without much concern about the potential misuses of the data collected and the overall ethical and legal underpinnings of these operations. This article examines three cases in point. The first case investigates the United Nations High Commissioner for Refugees’ decision to deploy a biometric matching engine engaging artificial intelligence to make accessing identification documents easier for both refugees and asylum seekers and the states and organizations they interact with. The second case focuses on the New Zealand government’s introduction of artificial intelligence to improve border security and streamline immigration. The third case looks at data scraping and biometric recognition tools implemented by the United States government to track (and eventually deport) undocumented migrants. The article first shows how states and international organizations are increasingly turning to artificial intelligence tools to support the implementation of their immigration policies and programs. Subsequently, the article also outlines how even despite well-intentioned efforts, the decision to use artificial intelligence tools to increase efficiency and support the implementation of migration or asylum management policies and programs often involves jeopardizing or altogether sacrificing individuals’ human rights, including privacy and security, and raises concerns about vulnerability and transparency.

## Introduction

In an increasingly globalized world, innovative technologies have come to offer greater virtual connectivity. At the same time, globalization itself has brought with it an increase in physical connectivity through international migration (Switzerland, IOM UN Migration, 2019, p. 9). Together, the increase in innovative technologies and the movement of people across borders have driven states to reconsider how they manage migration. Today, states and international organizations deploying artificial intelligence in migration management play a central role in the definition of migrants’ identity and therefore, the opportunities, protection and assistance they can access. In other words, migration is increasingly becoming a transaction requiring migrants to exchange biometric and biographic data for access to resources or a jurisdiction. This transaction can be viewed as one where the migrant trades parts of their physical identity to be viewed as a person: an eye for an ‘I’.

The purpose of this article is twofold. First, I argue that states and international organizations are increasingly turning to artificial intelligence as a tool to support the implementation of their migration or asylum management policies and programs. Such policies and programs may have very different goals; they may aim to restrict movement and control migration or access to asylum; they may seek to speed up the processing of economic migration or to support enforcement inland, but they may also aim to provide more efficient assistance to asylum seekers. The common element in these very different goals is the search for efficiency. This article assesses three cases in which various artificial intelligence (AI) capabilities are introduced to implement migration policies and programs relating to specific populations. I have selected these three cases due to the varying AI tools used, the different populations that interact with or are targeted by the tools and finally, the variance amongst the organization or states’ views of immigrants.

First, I assess the United Nations High Commissioner for Refugees’ (UNHCR) introduction of biometric data collection using iris and fingerprint recognition technology paired with Accenture’s Biometric Matching Engine to ensure that refugees “receive assistance where and when they need it” (Lodinová, 2016, p. 95). Then, I turn to New Zealand, where operational algorithms are used to avoid unnecessary burden in the country’s external border controls generally. Here, I particularly focus on four use cases: biometric and biographic matching, customer segmentation based on risk, customer screening based on eligibility, alerts, watchlists and risk and finally for case prioritization. I then analyze how the United States (the U.S.) pairs its biometric database, face and iris matching algorithms, fingerprint matching technology, the Investigative Case Management system and data scraping technology to track, locate and ultimately drive the deportation of undocumented migrants.

Much of the existing literature that assesses the use of innovative technologies in the migration sector cautions against the use of AI technologies, particularly due to their current shortcomings. Accordingly, this article’s secondary purpose is to contribute to the existing literature by showing how, even despite well-intentioned efforts, the decision to use AI as a tool to increase efficiency and support the implementation of migration or asylum management policies and programs often involves jeopardizing or altogether sacrificing individuals’ human rights, including to privacy and security, and raises concerns about vulnerability and transparency.

The article starts with an introduction to relevant technical concepts. I provide an introductory explanation of these concepts upfront to offer the reader an organized space to return to as they navigate the article. Then, I assess the use of AI to manage migration by the UNHCR, New Zealand and the U.S. In each case, I outline the state or organization’s intention and whether it is pro- or anti-immigration, explain the use case and offer a cursory technical assessment of the technologies deployed. I then offer a comparative perspective on the efficiency, security, transparency and vulnerability and human rights aspect of each case. Finally, I discuss how, in introducing AI to manage migration, efficiency is often exchanged for human rights protection.

## Clarifying key concepts

Artificial intelligence has become a buzzword in many areas of governance whether it concerns the future of work, public administration, security or indeed addressing emergencies like the COVID-19 pandemic. Below, I start by clarifying some key terms and concepts that are used both by experts and in common parlance with a view to setting the framework within which this paper is situated and introducing some of the key concerns that will be discussed in the following sections with specific reference to migration and asylum governance.

### Big data

The term big data refers to data, both qualitative and quantitative, that is too large, fast or complex to process using traditional methods (SAS, 2021). While big data and data management are often described by various ‘V’ words, five are important to know to gain a better understanding of what is meant by ‘big’ data: volume, velocity, variety, variability and veracity (Firican, 2017). Volume is the amount of information produced from sources—things such as business transactions, IoT devices, videos and social media. Velocity refers to the speed of the cycle at which data is produced, used and managed. Variety refers to the different formats that data can exist in, including structured and unstructured. Structured data is numeric data saved in traditional databases, while unstructured data includes emails, audio, videos, financial transactions and text documents. Additionally, variability refers to the varieties of data and data sources, while veracity refers to the quality of data, based on the ability to link, match, cleanse and transform information. Big data, which can be acquired through public and private sources, is what machines use to learn.

### Artificial intelligence

Artificial intelligence, or AI, refers to "machine-based operations that mimic human intelligence” (Schmidt & Stephens, 2019, 133). There is currently no clear definition of AI (Scherer, 2016, 359). Late AI pioneer, John McCarthy credits this to the challenge of how humans view intelligence, particularly, because “we cannot yet characterize in general what kinds of computational procedures one wants to call intelligent” (2007, 3). It is helpful to view intelligence as a property that something can have: humans can be intelligent, and so too, can machines. Nevertheless, AI is viewed as an unfit way to describe the capabilities of current technologies, which only provide one component of intelligence—prediction (Agarwal et al., 2018, 3).

### Machine learning

To understand prediction in AI, an introductory understanding of machine learning is required. Machine learning is a subset of AI where applications ‘learn’ from big data and, over time, improve their accuracy. Machine learning automates analytical modelling and can occur in four ways: supervised learning, unsupervised learning, semi-supervised learning and reinforcement machine learning (Lorberfeld, 2019). While exploring the methods a machine uses to learn is outside the scope of this  article, it is important to understand that machine learning largely depends on the algorithm used (Lorberfeld, 2019). Supervised learning engages a training dataset, which includes both an input and output variable, and uses a mathematical equation (an algorithm) to map a path from the input variable to the output variable. Gradually, the machine learns from the training dataset so when new input data is introduced, it can predict what the output variable is. Supervised machine learning includes two subcategories: classification and regression (Agarwal et al., 2018, 101). While both classification and regression rely on training datasets, under classification, machines learn to assign the input data as observations in categories, whereas under regression, the output of the above function is a numerical figure (Garbade, 2018). Conversely, unsupervised machine learning finds patterns or mapping functions where the output variable is unavailable. Here, the machine uses the algorithm to discover interesting structures in the data (Brownlee, 2016). Unsupervised learning includes two subcategories: clustering, which involves grouping observations based on similarities, and association, which involves discovering rules that describe relationships that characterize large sections of the data.

### Predictive analytics

Both supervised and unsupervised machine learning use prediction. Prediction is “the process of filling in missing information” using information that exists to generate information that does not exist (Agarwal et al., 2018, 13). Predictive analytics is an area in statistical science that extracts existing data and processes it to forecast—or make predictions about—trends and outcome patterns. Predictive analytics, unlike machine learning, is heavily informed by statistics. The more a machine learns, the more data it collects and, subsequently, by training on this data, the machine becomes better at making predictions. Today, everything can be seen as a prediction problem: taking information that one has about a problem and using it to discover more. This is exemplified by language translation: once a task for a linguist, is today, a task for Google Translate. To be clear, however, prediction does not equate to understanding.

### Deep learning and neural networks

Deep learning, a branch of machine learning, allows computers to perform human tasks. Deep learning uses algorithms inspired by the structure and function of the human brain (Brownlee, 2016), called “artificial neural networks” to undertake both supervised and unsupervised learning methods and progressively develop a better understanding of the relationship between the input and output data*.* Like how humans learn gradually, in deep learning, algorithms are used to repeat a task over time, with a slight change being made to improve the process and subsequently improve the output each time (Marr, 2018). Deep learning is used in processes such as facial and iris recognition, where "algorithms recognize edges at a certain level, nose at another level and face at yet another level” (Wu, 2019). To do this, deep learning requires big data to learn from.

### Black and white boxes

AI can sometimes present a particular challenge in that it may engage a “black box” system, which involves a machine learning model where it is difficult or altogether impossible to understand what the machine is doing when it is learning (Kuang, 2017; Bathaee, 2018, p. 905). Black boxes can be either strong or weak. In strong black boxes, humans are unable to understand how the AI arrived at a decision, prediction or output, what the system prioritized in determining the outcome and how it ranked the importance of variables. While the decision-making process in a weak black box is also opaque, the system can be probed and an explanation of how the AI ranked the importance of the variables it considered can be made apparent, which subsequently may offer the limited ability to speculate how the system arrived at its decision (Bathaee, 2018, p. 906). White-box models are completely interpretable, where their behaviour, how they produce predictions and what variables influence decision-making are entirely discernable. Two key elements of a white-box model are features that are understandable and a machine learning process that is transparent (Sciforce, 2020).

### Intention scale for the use of AI to deliver immigration policies and programs

**Fig. 1:**
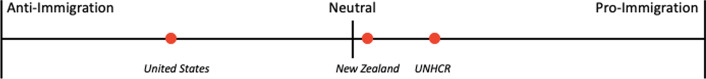
Intention scale for the use of AI to deliver immigration policies

To approach the consideration of each case against one another and critically analyze the underpinnings of AI use in migration and asylum management more specifically, it is helpful to think of each of the following cases on a sliding scale that captures the intentions behind the use of AI to deliver an immigration policy or program (see Fig. 1). This is an important component of conversations surrounding efficiency, security, legality, fairness, vulnerability and human rights concerning the use of AI tools.

### Impact and vulnerability scale for the use of AI to manage migration

**Fig. 2:**
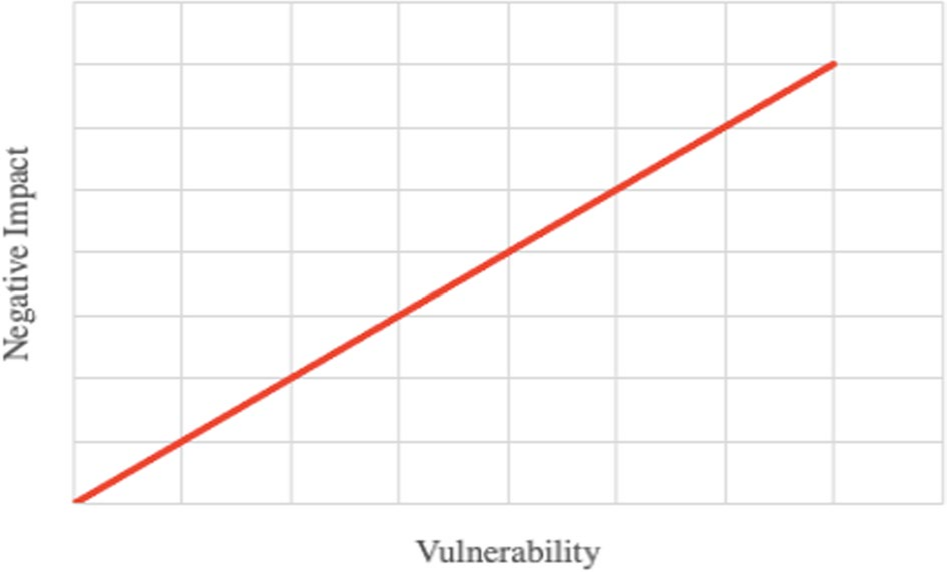
Impact and vulnerability scale for the use of artificial intelligence to manage migration

To analyze the relationship between the impact of using AI to manage migration and the vulnerability of the intended user or target group, it is helpful to envision a matrix that shows the more vulnerable the migrant (for example, refugees and asylum seekers), the more detrimental the use of AI in migration processes will be. Similarly, the less vulnerable the migrant, the less detrimental the use of AI (see Fig. 2). It should be noted that each migrant's journey, regardless of the purpose of migration, is unique and can make them vulnerable.

While the cases selected differ in terms of who develops and implements the AI tools, with what intention and on which populations, I believe they serve the purposes of my comparison because they illustrate the risks involved in using AI precisely in such different settings. To facilitate a logical comparison, I have organized each case study in three sections: I first consider the intention of the use of advanced digital technologies for addressing a governance issue; second, I analyze the technology and how it is used; and third, I offer a technical assessment that discusses the relationship between the AI technology used and the vulnerability of the relevant population.

## United Nations High Commissioner for Refugees

### Intention

According to the UNHCR’s mission statement, the international organization’s “primary purpose is to safeguard the rights and well-being of refugees. In its efforts to achieve this objective, the UNHCR strives to ensure that everyone can exercise the right to seek asylum and find safe refuge in another state, and to return home voluntarily. By assisting refugees to return to their own country or to settle permanently in another country, the UNHCR also seeks lasting solutions to their plight” (UNHCR, 2007). In particular, the UNHCR’s intention to assist refugees to return home or find refuge in a different country shows that the organization is pro-migration and therefore, aims to implement pro-migration policies. The UNHCR posits that a particular challenge in delivering services to refugees and asylum seekers, for both the organization itself and states which receive refugees, is the need to confirm their identity (UNHCR, 1984). Accordingly, to increase efficiency by streamlining service delivery and administrative tasks, the UNHCR has procured and deployed technologies, including AI, to ensure that refugees and asylum seekers not only have identification documents but that they are easily accessible for both the individual and the states and organizations they interact with.

### The use of AI

At the centre of the UNHCR’s effort to ensure that refugees and asylum seekers have easily accessible identification documents is the UNHCR’s digital Population Registration and Identity Management Ecosystem (PRIMES). “PRIMES brings together all of the UNHCR’s digital registration, identity management and case management tools into one internally connected and interoperable ecosystem” (UNHCR, n.d.). PRIMES includes several different repositories of personal data, both biographic and biometric, and enables identity management and documentation, case management and assistance (both cash and in-kind). According to the UNHCR, “PRIMES has been designed with cooperation and interoperability in mind so that refugee supporting governments and partners can make use of the data and technology which UNHCR has developed to enhance their ability to deliver services often together with the UNHCR, in a safe and secure manner. PRIMES is fully aligned with the Policy on the Protection of Personal Data of Persons of Concern to UNHCR” (UNHCR, n.d.).

**Fig. 3:**
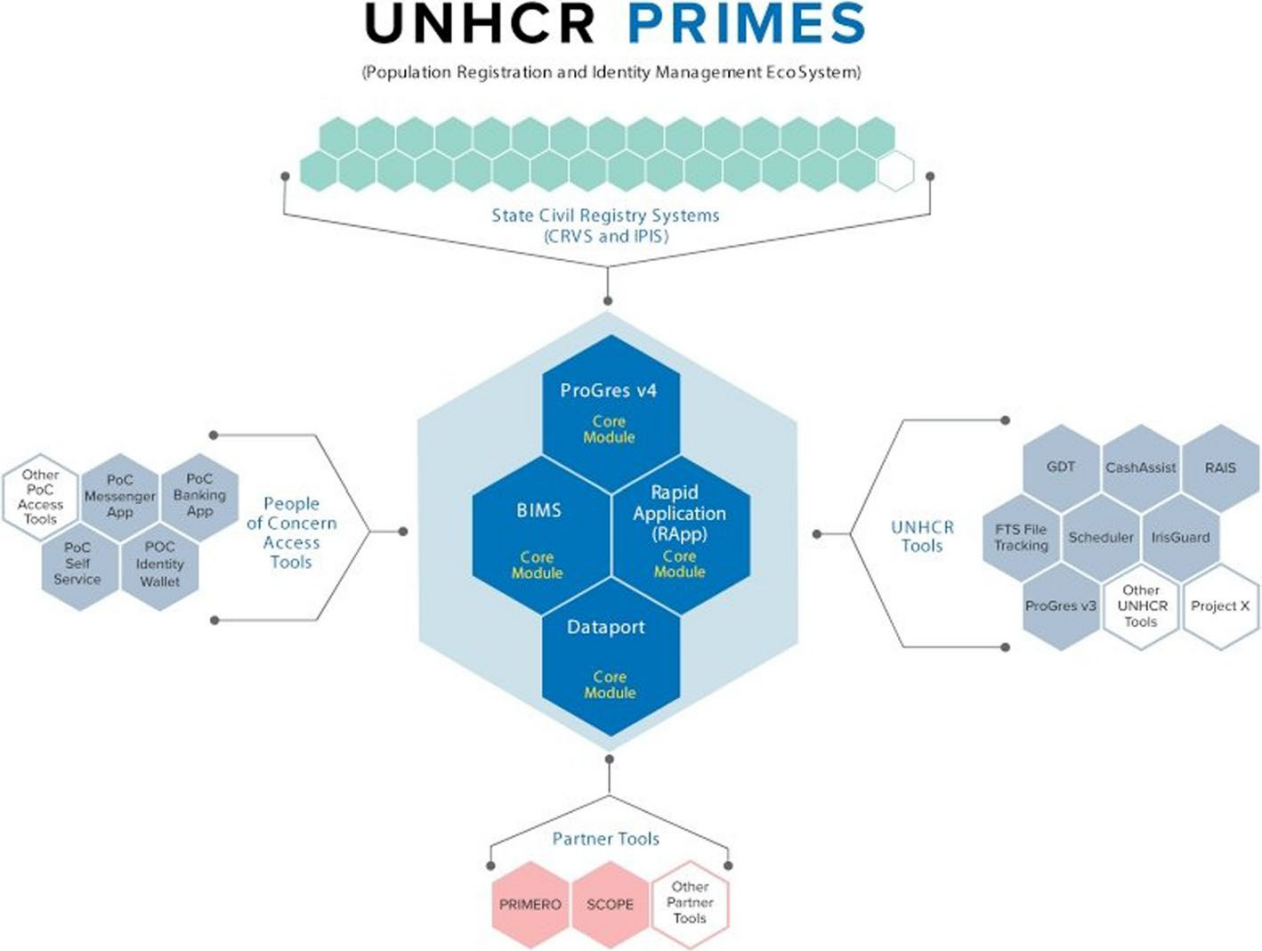
UNHCR's Population Registration and Identity Management EcoSystem (PRIMES) (*Source*: UNHCR, n.d.)

Among the tools housed under PRIMES is the UNHCR’s primary biometric identity management system (BIMS) (see Fig. 3). Released in 2015, BIMS was developed by Accenture and engages Accenture’s Unique Identity Service Platform, which uses an individual’s ten fingerprints and two irises to build a globally available biometric record (Sánchez-Monedero, 2018, p. 7). Part of Accenture’s Unique Identity Service Platform includes the Biometric Matching Engine, which is a software that uses AI to “compare a variety of biometric identifiers, such as face, fingerprints, iris or voice, against large volumes of reference identity data. Based on this information, the software automatically creates a unique identification strategy to optimize the speed and effectiveness of database search for a matching record” (Security Document World, 2016). BIMS, which is deployed globally, allows an individual to present two or more biometric elements (any combination of eyes and fingers) for their identity to be ascertained in a matter of seconds (Sánchez-Monedero, 2018, p. 7).

Accenture’s Biometric Matching Engine employs a patented “dynamic matching strategy,” which identifies and contrasts individual records within databases comprising of at least two sets of biometric modalities (Security Document World, 2016). According to the patent, the system works by “receiving, by a processing device, a first input biometric sample and a second input biometric sample; determining, by the processing device, a first characteristic associated with the first input biometric sample and a second characteristic associated with the second input biometric sample; grouping, by the processing device and after receiving the first input biometric sample and the second input biometric sample, a plurality of records to form a first set of records and a second set of records, the grouping being based on the first characteristic and the second characteristic; performing, by the processing device and on the first set of records, a first matching process based on a first threshold; performing, by the processing device and on the second set of records, a second matching process based on a second threshold; and identifying, by the processing device, a record, of the plurality of records, based on a result of the first matching process and a result of the second matching process” (Security Document World, 2016).

### Technical assessment

In the above case of the UNHCR, I focus on the interaction of two technologies in PRIMES. First, I focus on biometric identifiers, like the iris and fingertips, then, I focus on BIMS and the Biometric Matching Engine, which identifies and compares biometric records in a large database of biometric records. To assess the BIMS and Biometric Matching Engine, it is important to understand the strengths and shortcomings of biometric recognition technologies, specifically in this context, iris and fingerprint scanners. Biometric recognition involves studying an individual’s unique physical traits and behavioural characteristics (Umer et al., 2017, p. 1). In the field of biometric recognition, iris scans offer a particular advantage in that they are accessible and involve the use of a unique physical trait that is claimed to be stable over an individual’s lifetime. No two irises are alike, not even those of twins (Umer et al., 2017, p. 1). The likelihood of one’s iris being affected by damage is low, as it is protected behind the eyelid and the cornea. Iris recognition is an automated method of biometric identification that employs mathematical pattern-recognition techniques on an image of the eye to identify an individual. Biometric iris scanners first illuminate the iris with invisible infrared light, picking up the iris’ unique patterns. The result is a set of pixels that only contain the iris, including information collected from roughly 240 biometric features (Electronic Frontier Foundation, n.d.). The pixels are then analyzed and a bit pattern is extracted which encodes the information in the iris. The bit pattern is digitized and stored in a database for verification or identification. Although iris scans offer the advantage of being consistent and unique throughout an individual’s life, the capability is not without its shortcomings. While the technology is arguably one of the more reliable biometric identification methods, it has the potential to yield negative or inaccurate results due to long eyelashes, contact lenses or watery eyes (Mayhew, 2012). Furthermore, there have been reports suggesting the claim that the iris does not change throughout an individual’s life is unreliable (Baker et al., 2012). If a database of information is stolen or compromised, this can have detrimental effects, especially given individuals cannot simply get a new set of irises. This issue is further exacerbated by the fact that iris biometrics are usually collected and stored by third-party vendors (Electronic Frontier Foundation, n.d), for whom data privacy and the safety and security of migrants may not be a top priority. Furthermore, in the above-described case, the iris data collected and stored is that of refugees and asylum seekers, which is particularly important to note, as a breach of the database may lead to the discrimination, involuntary repatriation, resettlement or further persecution of these vulnerable individuals (Madianou, 2019, p. 590–591).

In contrast, while four types of fingerprint scanners exist, they all work by collecting the digital image of the surface of a fingerprint and finding a match for it in a database. The most common fingerprint matching techniques are pattern matching and minutiae-matching. The former compares two images to determine how similar they are, while the latter relies on the specific location and direction of points in a fingerprint referred to as ‘minutiae’ (Mayhew, n.d.). Like iris scans, fingerprint scans present security issues, as they are easily replicated through photographs and 3D printing. Fingerprints, more commonly than irises, can be burned or damaged due to abrasion and are, therefore, arguably unstable.

Accenture’s Biometric Matching Engine, which employs a patented “dynamic matching strategy,” identifies and contrasts individual records within databases. In the context of the UNHCR, the Biometric Matching Engine engages the biometric data of refugees and asylum seekers, including iris and fingerprint scans, to locate matches in a database. The BIMS “links verification stations in UNHCR offices and camps around the world back to a central biometric database in Geneva” (Accenture, 2015).

While I argue that the intention with which the UNHCR uses biometric recognition technology is to implement pro-immigration policies, it would be negligent to not acknowledge that the organization may grant the technological capabilities more credit than they deserve. For example, Mirca Madianou explains that the United Nations has been using iris scanning technology since 2002, during the repatriation of more than 1.5 million Afghan refugees from Pakistan (2019: 588). In this case, the technology was used to identify individuals who sought funds “more than once” (Kessler, 2002). According to Madianou, “if an algorithm detected that a new entry matched an already existing iris record, the claimant was refused aid. The UNHCR representative in that mission declared his trust in iris technology when he stated that as a result, decisions could no longer be disputed: ‘how can [refugees] argue now, the machine can’t make a mistake’” (2019, p. 588). From March to October 2002, 396,000 false claimants were turned away from receiving aid (Kessler, 2002). However, iris recognition hosts an error rate of 2–3%, which, as Madianou shows, suggests 11,800 claimants out of the alleged false claimants were wrongly denied aid (2019, p. 588).

When considered on the Impact and Vulnerability Scale (Fig. 2), the challenges of biometric recognition technologies can hurt migrants. However, because in the above-described example these technologies are deployed in the management of migrants who are vulnerable, particularly refugees and asylum seekers, this technology can have an especially detrimental impact on these groups. Accordingly, the above-described example shows that despite an organization’s intentions, the shortcomings and risks of AI capabilities can lead to unintended negative implications for migrants, including refugees and asylum seekers.

## New Zealand

### Intention

According to Immigration New Zealand, the government department that manages migration and border control in New Zealand, “[the organization] work[s] with international organisations and industry partners to improve border security and make immigration easier. [The organization] lead[s] government strategy designed to help migrants settle in New Zealand” (Immigration New Zealand, n.d.) The government of New Zealand, through Immigration New Zealand (Immigration NZ) aims “to manage risk to New Zealand and ensure that travellers pass through [its] borders and receive immigration decisions quickly consistent with Government policy” (Stats NZ, 2018b, p. 33). New Zealand’s intention to manage risk while ensuring good service delivery for travellers shows that the organization is pro-migration, but not as centrally focused on assisting immigrants in travelling as the case of the UNHCR. The following describes how Immigration NZ has introduced algorithms to manage risk while improving service delivery for immigrants, together denoting a desire for increased efficiency.

### The use of AI

Data modelling and algorithms have been introduced to manage migration in New Zealand since 2014 (Stats NZ, 2018a, p. 18). While there was speculation that a short-lived attempt was made to use algorithms to predict which immigration visa applicants would cost the state financially (Bonnett, 2018), the use of AI in migration management in New Zealand has mostly focused on managing overall traffic through the country’s borders rather than immigration more specifically.

The first introduction of AI was speculated to be an experimental tool, specifically, a data modelling pilot programme that came into use in April 2018. The programme, referred to as a “harm model,” was developed and being used in Immigration NZ’s Auckland office (Dynon, 2019). The system reportedly used Microsoft Excel spreadsheets (Pullar-Strecker, 2018a) to analyze historical data, including age, gender, country of origin, visa held upon entering New Zealand, involvement with law enforcement and health service usage of 11,000 individuals overstaying their visas (Pullar-Strecker & Reidy, 2018). Other variables considered included unpaid hospital debts, number of failed immigration applications and immigration fraud allegations (Bonnett, 2018). According to Alistair Murray, Immigration NZ Compliance and Investigations Area Manager, this practice involved “predicting how someone is most likely to behave based on how their predecessors have behaved” (Fraser, 2018). The information was used to forecast the financial impact each overstayer could have in the future, or, in other words, how much each irregular immigrant would cost New Zealand (Bonnett, 2018). Murray further explained that this “information [would be coupled with data] from the other data sets and then [the government looked] at building a profile of the type of person that [it] want[ed] to remove at the earliest possible opportunity” (Fraser, 2018). This information would subsequently be used to prioritize deportation over prosecution or allowing immigrants to reapply for a visa (Tan, 2018).

The aforementioned example has often been cited in relation to the use of innovative technologies to manage migration, however it is unclear if and how this project deployed AI, and in any case, this project was short-lived, as then Immigration Minister Lees-Galloway asked Immigration NZ to suspend the practice until it was discussed with the Human Rights Commission and Privacy Commissioner, John Edwards (Pullar-Strecker, 2018a). In an algorithm assessment document, Immigration NZ indicated that the compliance resources that were allocated for the programme were re-focused on another issue and the project therefore did not progress (Stats NZ, 2018b, p. 41). The aforementioned case, while having received significant criticism (Tan, 2018), drove the New Zealand government to order a stock-take of how algorithms were being used to crunch people’s data (Pullar-Strecker, 2018b; Stats NZ, 2018b) and experts to call for the government to provide more systematic transparency on if and how it was experimenting and deploying algorithms (Gavaghan et al., 2019). In October 2018, the New Zealand government published the Algorithm Assessment Report, which described the use of ‘operational algorithms’ in managing migration to New Zealand (Stats NZ, 2018a). This report brought to the surface some interesting insights on the management of NZ’s borders.

According to Immigration NZ, “the volume of transactions handled by [Immigration NZ] is large and growing. [Immigration NZ] in 2016/17 made 800,000 immigration decisions, covering over a million people. Over 6,750,000 travellers passed through New Zealand’s borders in 2016/17. Data and algorithms assist with risk management and managing this volume of transactions effectively” (Stats NZ, 2018b, p. 33). Immigration NZ uses algorithms in four particular ways: biometric and biographic matching, customer segmentation based on risk, customer screening based on eligibility/alerts/watchlists/risk (for example, Interpol alerts) and for case prioritization (Stats NZ, 2018b, p. 34). In all instances, it remains unclear whether the technology is used specifically for visitors, immigrants or both. Biometric and biographic matching take place in Immigration NZ’s Identity Management System, where data including pictures of faces or fingerprints (biometric data) and biographic data like birth dates, marriage, addresses are matched to an individual using an operational algorithm (Stats NZ, 2018b, p. 34). The system was developed and delivered by Datacom with support from external technology providers (DAON, NEC and intech), who continue to provide support for the system (Stats NZ, 2018b, p. 37). The information held in the system can be used to assist in decisions relating to the visa decision process, as deciding on an individual’s identity is part of the visa process. Notably, because of the strict threshold configurations of the Identity Management System, roughly 35% of all cases that are matched require assistance from a human to perform a manual identity resolution (Stats NZ, 2018b, p. 34). The management and oversight of the Identity Management System is twofold; the first level oversight group is the Triage Reference Group, which decides on thresholds, methodologies and risk rules, while the Immigration NZ Operating System Integrity Committee acts as a second level governance group.

Customer segmentation based on risk is part of the visa application risk triage process where an application is assessed and decided upon. In particular, the software assigns a risk level to visa applicants based on risk rules that use information that Immigration NZ has about the applicant. The risk level acts as a guide for the level of verification that an immigration officer must perform subsequently and has no direct relationship with the final decision (Stats NZ, 2018a, p. 17; Stats NZ, 2018b, p. 35). The rules were developed by subject matter experts, with the help of external providers, including SAS Business Analytics Advisory Services, PricewaterhouseCoopers, Dragonfly and Knoware (Stats NZ, 2018b, p. 36). Assessments use both qualitative and quantitative data. In this case, the risk rules originate from statistical modelling and are mostly used to identify low-risk applicants. The rules are generated based on modelling of past decisions and to be used, require a high level of statistical confidence. While the final decision to grant or decline a visa remains with the immigration officer, the customer segmentation process helps streamline visa processing as it offers a guide for verification each application requires based on the risk level assigned to it by the system.

The operational algorithms used in customer screening based on eligibility, alerts, watchlists and risk occur in the travel phase of a customer’s journey. This process occurs through the Advance Passenger Processing system, which collects data provided by individuals to airlines during check-in, including information from their passports. The Advance Passenger Processing system then uses algorithms to automate the validation of the data and conduct border checks, including confirming the validity of visas and matching passports to a list of lost or stolen passports. Depending on the results of the aforementioned automated checks, passengers may be denied boarding (Stats NZ, 2018b, p. 36). Finally, operational algorithms are used in screening customers based on risk. In this instance, a set of risk rules are developed by subject matter experts. These rules are subsequently used to perform border checks offshore, where foreign nationals travelling to New Zealand embark on their trip. Airlines provide a list of passengers to New Zealand through a computer system which is then evaluated alongside Passenger Name Records, by immigration staff who use the risk rules to manually search for abnormalities or patterns. This programme is called the Risk Targeting Programme and it combines data from the Advance Passenger Processing system and Passenger Name Records (Stats NZ, 2018b, p. 36).

The majority of the algorithms Immigration NZ uses were developed by internal staff and employ analytical tools to analyze big data sets and identify patterns and trends. While the New Zealand Government Chief Data Steward, Liz MacPherson, says that the above does not count as AI (Kenny, 2018), the Data Ethics Advisory Group in August 2020 raised concerns captured in a feedback form that “training of the data models (machine learning) relies on past data and that Immigration NZ’s dependence on this could have implications for the operations of the Risk Analytics Platform [sic]” (New Zealand Government Chief Data Steward, n.d.) indicating that the algorithms being used are in fact machine learning algorithms.

### Technical assessment

According to Stats NZ’s Algorithm Assessment Report, the algorithms used by Immigration NZ are operational algorithms (2018a: 16), which it defines as “analytical processes [that] interpret or evaluate information (often using large or complex data sets) that result in, or materially inform, decisions that impact significantly on individuals or groups. They may use personal information about the individuals or groups concerned, but do not need to do so exclusively” (Stats NZ, 2018a, p. 7). Gavaghan et al. provide a more technical definition of ‘operational algorithms,’ specifically, “predictive models [which] are models which make predictions about some unknown variable, based on one or more known variables” (2019, p. 6). To build a predictive model, data is necessary to both train the model and to use it. As indicated by Immigration NZ itself, “the volume of transactions handled by [Immigration NZ] is large and growing” (Stats NZ, 2018b, p. 33).

To this end, pictures of faces or fingerprints (biometric data) and biographic data like birth dates, marriage, addresses and flight data. The use of this data and algorithms and predictive models present some challenges, many of which Immigration NZ already recognizes (Stats NZ, 2018a,p. 7–8). The output of an algorithm reflects the data it draws upon. Therefore, poor data and data management will hinder the accuracy and predictive ability of the algorithm (Stats NZ, 2018a, p. 7). Furthermore, the data the algorithms are trained on may have inherited human biases and errors which can result in the algorithm applying those same errors and biases in the analytical process. This, in particular, is a notable shortcoming of the use of algorithms in managing migration because algorithms that have inherited human biases and errors can further reinforce inequality.

Notably, while it is unclear what specific technology underpins the predictive analytics used by Immigration NZ, there are predictive models wherein it can be difficult if not entirely impossible to understand what the algorithm is doing. In these systems, referred to as “black box” systems, humans may have difficulty in or may altogether be unable to understand how the system arrived at a decision, prediction or output, what the system deemed essential in determining the outcome, and how the system ranked the variables it processed in order of importance. To avoid making decisions in the dark, explainable models are often preferred. However, explainability does not equate to intelligibility, or rather, the ability to comprehend the explanation of how a model arrived at the outcome (Gavaghan et al., 2019, p. 41). Notably, often there is a trade-off between a system’s explainability and its predictive performance (Gavaghan et al., 2019, p. 10). Furthermore, as is often the case with technological solutions, primarily those that involve emerging technologies like AI, even if explainability is not a challenge, transparency and accessibility may be. Gavaghan et al. explain that “Intellectual property rights might prevent the disclosure of proprietary code, or preclude access to training data, so that even if it were possible to understand how an algorithm operated, a full reckoning may not be possible for economic, legal or political reasons. Algorithms that are otherwise technically transparent may therefore be “opaque” for nontechnical reasons” (2018, p. 41).

Because these technologies are used to establish an individuals’ identity, assign a risk level to a visa applicant, and grant or deny migrants the ability to travel to the country, the impact of these technologies has the potential to be detrimental. Given that it is unclear whether predictive models are applied to or target vulnerable groups,^1^ when considered on the Impact and Vulnerability Scale (Fig. 2), the challenges of predictive models, or as Immigration NZ calls them, ‘operational algorithms,’ can present a negative, substantially negative or entirely detrimental impact on migrants. The above-described example shows that despite having good intentions, states that are generally pro-migration and seeking to strike a balance between security and efficiency can fall victim to the allure of AI, where the shortcomings of AI capabilities can lead to unintended negative consequences for migrants.

## The United States

### Intention

To assess the intention of the U.S. in developing and implementing its immigration policies, I considered the missions of U.S. Immigration and Customs Enforcement (ICE) and U.S. Citizenship and Immigration Services. The purpose for this twofold consideration was that while U.S. Citizenship and Immigration Services is responsible for administering the state’s immigration system, immigration enforcement is the responsibility of ICE.

The mission of U.S. Citizenship and Immigration Services is to “[administer] the nation’s lawful immigration system, safeguarding its integrity and promise by efficiently and fairly adjudicating requests for immigration benefits while protecting Americans, securing the homeland, and honoring our values [sic]” (U.S. Citizenship & Immigration Services, n.d.). How U.S. Citizenship and Immigration Services positions the U.S.’s immigration system as one that is entrenched in the law and for which integrity must be safeguarded is defensive. The statement centres around the country as it exists, without any mention of migrants.

ICE’s mission is “to protect America from the cross-border crime and illegal immigration that threaten national security and public safety” (U.S. Department of Homeland Security, n.d.). ICE describes itself as follows: “ICE stands at the forefront of our nation's efforts to strengthen border security and prevent the illegal movement of people, goods, and funds into, within, and out of the U.S. The agency's broad investigative authorities are directly related to our country's ongoing efforts to combat terrorism at home and abroad” (U.S. Immigration and Customs Enforcement, n.d.). While the approach that ICE takes to describe its mission and purpose is like U.S. Citizenship and Immigration Services in that it is defensive, the mission itself much more blatantly positions the U.S. as a nation that requires safeguarding and security against foreign threats. Together both Departments' intentions appear to be focused on defence and security, prioritizing border security and preventing the irregular movement of people, without much regard for immigrants otherwise. Accordingly, the U.S. intentions are anti-immigration. What follows is a description of the players and technologies the U.S. engages to efficiently enforce their anti-immigration policies.

### The use of AI

**Fig. 4:**
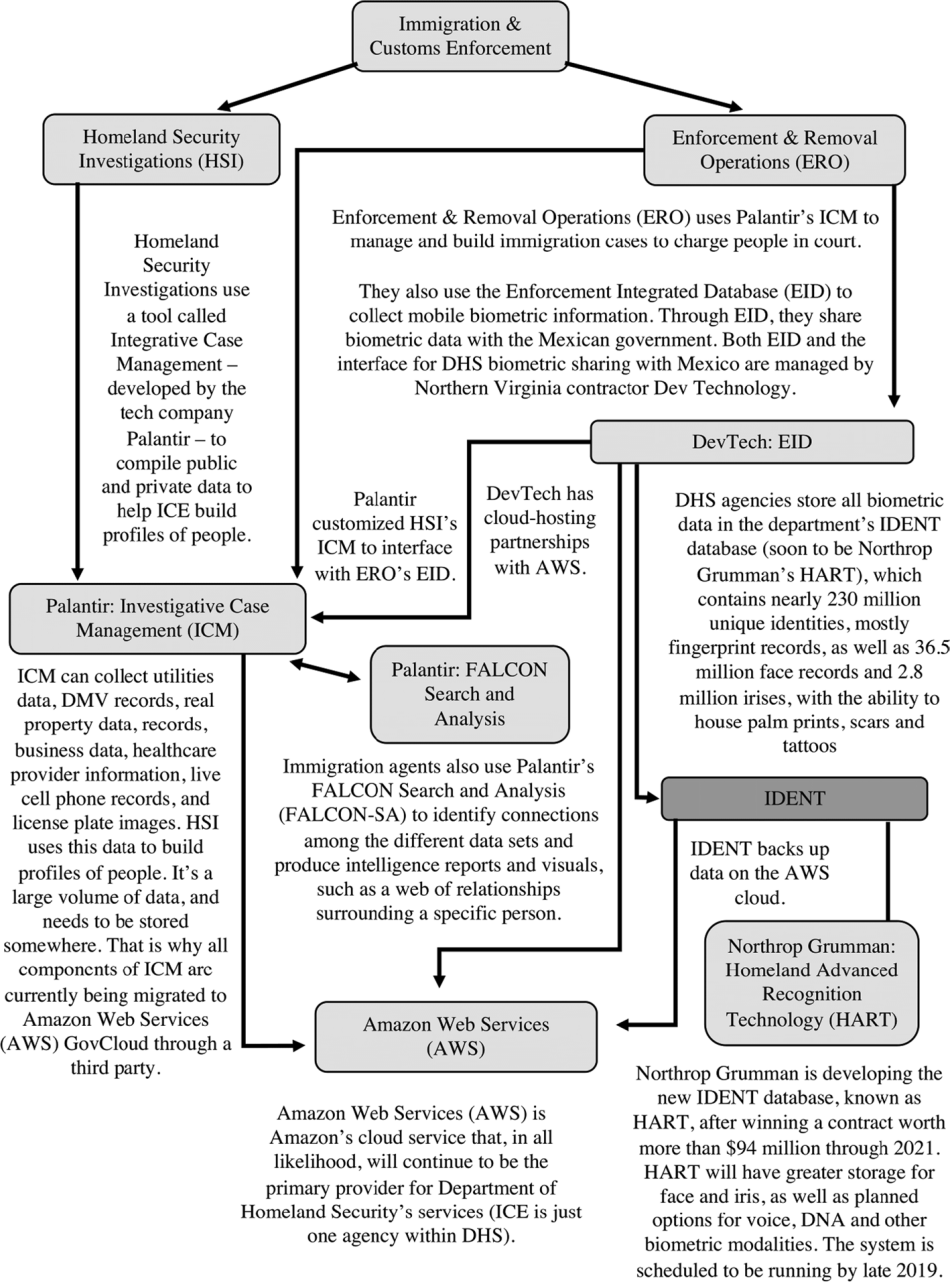
Tech Suppliers in U.S. ICE Operations (*Source*: Investigate, 2,018)

Immigration and Latinx-focused organizations working at the intersection of migration, technology and policing, together commissioned Empower LLC, a research company focused on improving corporate transparency, to research the technologies being deployed by the U.S. government to manage migration. Through its investigation, Empower LLC discovered that while there have been a multitude of private companies involved in the use of data and AI in managing migration in the U.S. seven players are pivotal in the operation: Northrop Grumman, Palantir Technologies, Inc., Giant Oak, NEC Corporation, Gemalto, Thomson Reuters and Amazon. What follows is a description of these technology suppliers’ involvement in the management of migration in the U.S. (see Fig. 4).

In February 2018, Northrop Grumman was awarded a $95 million contract to develop the initial phases of the Department of Homeland Security’s (DHS) new biometric database called Homeland Advanced Recognition Technology (HART). HART’s predecessor is the Automated Biometric Identification System (IDENT), where biographic and biometric data, including fingerprints, palm prints, facial image and iris scans, and information on sex offenders, wanted criminals, deported felons, immigration violators, and individuals with criminal histories was previously stored (Investigate, 2018). HART, IDENT’s replacement, has the capacity of storing at least 500 million unique identities, including the biometric data, and “DNA, facial and voice recognition, scans, tattoos and ‘other modalities’ (Investigate, 2018). HART, like its predecessor, IDENT, will be hosted on the Amazon Web Services (AWS) GovCloud (Corrigan, 2019). This is where all of the big data is stored. To make use of this data, more specifically, match immigrants to the 500 million unique identities, face and iris matching algorithms developed by NEC Corporation and fingerprint matching technology from Gemalto will be used (Investigate, 2018). In addition to these technologies, Palantir Technologies Inc. was awarded a $51.6 million contract to develop the case management system that ICE uses to share information called the Investigative Case Management system. The Investigative Case Management system allows ICE agents to create and manage case files, by using information from a range of databases that are both internal and external to the DHS. Palantir also developed FALCON-SA (FALCON Search and Analysis), a software that allows ICE to search, analyze, visualize and most importantly, link, data from the Investigative Case Management system.

Although the DHS announced its plans to collect and study social media data on all immigrants in 2017, Giant Oak, a private firm that aims to find the “people behind the data” has been conducting this work for DHS since 2013. In particular, Giant Oak employs a deep web search engine titled Giant Oak Search Technology (GOST) to scrape publicly available data to extract biographic and geographic location information. GOST pairs behavioural science with machine-learning to identify keywords that may indicate an individual is engaging in criminal activities or reveals their immigration status (Investigate, 2018). More specifically, the algorithm may look for patterns in the physical places or online websites an individual visits. The algorithm learns when the user, usually investigators, provides the machine with feedback indicating whether the search result was relevant or not (Ravindranath, 2016).

Thomson Reuters, more specifically two subsidiaries of Thomson Reuters, Thomson Reuters Special Services and West Publishing Corporation have a contract with ICE’s Detention Compliance and Removal office for a system that provides real-time information and jail booking, cataloging arrested individuals’ vehicle registration information, insurance claims, credit history, payday loans, public court records, employer records, wire transfers and Taxpayer Identification Numbers. West Publishing, in particular, offers authorities access to public and proprietary information, including utilities data, Department of Motor Vehicles records, real property data, professional licenses, criminal and court records, information on individuals’ healthcare providers, their consumer and credit bureau information, incarceration and arrest records, business data, data from social networks, chatrooms and blogs (Investigate, 2018). Together, these technologies work to track, locate and ultimately drive the deportation of undocumented migrants.

### Technical assessment

The U.S. manages migration by collecting and using data: biographic and biometric data, including fingerprints, palm prints, facial image and iris scans, DNA, voice recognition, scans, tattoos, information on sex offenders, wanted criminals, deported felons, immigration violators, and individuals with criminal histories, social media data, including from social networks, chatrooms and blogs, incarceration and arrest records, including real-time information and jail booking, arrested individuals’ vehicle registration information, insurance claims, payday loans, public court records, employer records, wire transfers and Taxpayer Identification Numbers, utilities data, Department of Motor Vehicles records, real property data, professional licenses, information on individuals’ healthcare providers, credit history, including consumer and credit bureau information, business data. Recall the five Vs that I defined at the start of this article. The aforementioned data meet the qualification of “volume”—the amount of information produced from sources—things such as business transactions, IoT devices, videos and social media. Additionally, the variety of data here is noteworthy. Again, variety refers to the different formats that data can exist in, including structured and unstructured. Here, the data is unstructured, as it exists in various forms, including pictures, financial transactions and text documents. ICE searches, analyzes, visualizes and links this data to build profiles of people.

Additionally, publicly available data is scraped to extract biographic and geographic location information and behavioural science is paired with machine-learning to identify keywords that may indicate an individual is engaging in criminal activities or reveal their immigration status. Patterns are sought out in the physical places or online websites individuals visit to ultimately track, trace, seek out and deport migrants. The impact of this data collection and the deployment of these technologies is entirely detrimental for undocumented migrants. When considered on the Impact and Vulnerability Scale (Fig. 2), as there is not much, if anything, of the individual’s identity that the state has left private. The above-described case shows that states which have anti-immigration policies do not have much, if any, regard for irregular migrants’ rights, and therefore, are not particularly concerned with the negative consequences of AI on vulnerable communities.

## The trade-off between efficiency and human rights protection

The above-described examples use AI tools in various ways for different purposes, with the broader intent of migration management. As outlined throughout this article, the intention of each state or global organization is different, and those intentions are apparent in the immigration policies and programs they aim to implement. AI presents itself as a tool to support the implementation and delivery of these immigration policies and programs. In particular, with the UNHCR, despite an organization’s intentions, the shortcomings of AI capabilities can lead to unintended negative implications for migrants, including refugees and asylum seekers. Furthermore, the case of New Zealand showed that, despite having good intentions, states that are generally pro-migration and seeking to strike a balance between security and efficiency can fall victim to the allure of AI, where the shortcomings of AI capabilities can lead to unintended negative implications for migrants. Finally, as exemplified by the U.S., states with anti-immigration policies do not care much for irregular migrants’ rights and therefore the negative implications of AI technologies on vulnerable communities. While the establishment and assessment of the intention that drives the use of AI in each case shows a common concern for efficiency, across each case the balance between efficiency and protection largely depends on the intention of the state or organization.

The UNHCR introduced AI to reinforce refugees’ dignity, streamline services, reduce fraud, increase efficiency and centralize information (UNHCR, 2019). In comparison, the initial introduction of algorithms in New Zealand to prioritize the deportation of ‘harmful individuals’ while brief, set the wrong tone for the country. Importantly, however, the New Zealand government learned and acted quickly to re-route. This swift movement allowed New Zealand to develop transparency around the state’s use of AI in immigration and ultimately led to the New Zealand Government developing and subsequently releasing an Algorithm Charter, with the intention to demonstrate transparency and accountability in the use of data (Stats NZ, 2020). Finally, and most stark in contrast, is the offensive way in which the U.S. government uses AI to manage migration, invasively collecting biographic and biometric data to identify, track and ultimately deport ‘undesirable’ migrants.

Before an AI system is introduced into migration processes, four particular dimensions should be considered and assessed, including efficiency, security, transparency and vulnerability. Below, I describe some of these considerations concerning the UNHCR, New Zealand and the U.S. use of AI to manage migration. As evidenced by the cases assessed in this article, states and international organizations are increasingly turning to AI as a tool to support the implementation of migration or asylum management policies and programs. In doing so, the intentions of states and international organizations are driving their regard (or lack thereof) for individuals’ rights. To deploy AI to manage migration, efficiency is often exchanged for human rights protection.

### Efficiency

In all of the above cases, the UNHCR (Lee, 2016), New Zealand (Stats NZ, 2018a, p. 18) and the U.S. (Mijente, 2018, p. 36), efficiency is a central tenet of the introduction of AI tools. However, when compared to the intention of each state in deploying AI to manage migration, efficiency can be described differently. In the context of the UNHCR, aiming at assisting refugees, the use of AI is intended to streamline the number of documents that are required from refugees to identify themselves. While Emrys Schoemaker, Dina Baslan, Bryan Poll and Nicola Dell note that the biometric technologies have generally been well-received by refugees in camps, the tool is not well received by all, including Ugandan refugees, who were generally cognizant of the biometric system’s security abilities, likely because there had recently been a significantly large fraud scandal in Uganda’s aid distribution systems (2021, 21; Parker, 2018).

In New Zealand, AI is used to “manage risk to New Zealand and ensure that the approximately 6.7 million travellers who pass through the border annually receive speedy, consistent, immigration decisions” (Stats NZ, 2018a, p. 16). As indicated above, New Zealand uses operational algorithms in four particular areas, including biometric and biographic matching, customer segmentation based on risk, customer screening based on eligibility, alerts and watchlists and finally, case prioritization (Stats NZ, 2018a, p. 17). Generally, the applications of AI in New Zealand are neutral in that they do not seek to target a particular group efficiently. Rather, the “algorithms make travel through the border more efficient and less time-consuming for the majority of passengers” while simultaneously “process[ing] passengers travelling to New Zealand in advance, and support[ing] decisions related to the granting of a visa. This analytical support allows staff expertise to be targeted to areas of greatest need” (Stats NZ, 2018a, p. 18).

Conversely, the use of AI in the U.S. aims to efficiently track and target individuals based on the analysis of big data. In particular, this targeted profiling of individuals occurs in the case management portion of the system established by ICE (Mijente, 2018, p. 31). Developed by Palantir, FALCON-SA links data in the ICM. This information is subsequently used to identify, locate and deport individuals.

### Privacy and security

Similar to other personal data, there are security concerns surrounding the use and storage of data collected and used by AI tools. As Ana Beduschi argues, the centralized collection of this data is an attractive target for hackers (Beduschi, 2020, p. 7). One of the more concerning questions surrounding security is who owns the data that is collected and what, if anything, is done to ensure that it is protected. As Lilian Edwards and Michael Veale posit, “machine learning and big data analytics in general are fundamentally based around the idea of repurposing data, which is in principle contrary to the data protection principle that data should be collected for named and specific purposes” (Edwards & Veale, 2017, p. 18). Previously, the collection of this data was founded on principles of consent. However, consent has become nearly an illusion, particularly if, as the UNHCR case exemplifies so well, the subject whose information is being collected wants to be able to access any services. In the realm of AI and big data, issues of consent are heightened, as big data sets often include the use of inferred data which is collected from sources that have been consented to (Gavaghan et al., 2019, p. 46). A lack of transparency makes it difficult to ascertain what, if any, safeguards have been introduced to ensure the security of individuals’ private information. While the UNHCR has published information indicating that “PRIMES is fully aligned with the Policy on the Protection of Personal Data of Persons of Concern to UNHCR” (UNHCR, n.d.) this is a general statement and the organization does not explicitly outline how its technologies actually protect the personal data of persons of concern to the UNHCR.

While the New Zealand government has outlined in its Algorithm Charter its commitment to “regularly peer reviewing algorithms to assess for unintended consequences and act on this information,” (Stats NZ, 2020), it is unclear, which, if any of the aforementioned operational algorithms have undergone peer review and whether any changes have been made. Nor is it clear what the peer review process entails and whether it sufficiently addresses the importance of individuals’ privacy and security.

Finally, while the U.S. has conducted and updated privacy impact assessments on some of the technologies it engages, including the FALCON Search & Analysis System (U.S. Department of Homeland Security, 2016b) and the ICE Investigative Case Management system (U.S. Department of Homeland Security, 2016a), the assessments often favour the state and its law enforcement operations over individuals’ right to privacy.

### Transparency

All three of the abovementioned cases rank poorly for being transparent about the AI tools they have developed or procured and deployed in managing migration. The challenge of transparency is further exacerbated when algorithms and AI tools are procured or built by external suppliers, who subsequently refuse to reveal the development of the tools because the information is proprietary. While there are some mentions of the use of iris recognition technology and BIMS and the Biometric Matching Engine by the UNHCR, there is insufficient information describing the technologies in use. What makes the case of the UNHCR particularly frustrating is that the UNHCR, as an international organization, does not have a citizenry to whom it is accountable, and therefore, it becomes easier for the organization to fly under the radar of citizens’ scrutiny.

Furthermore, concerning New Zealand, transparency only came about after the media made it public that, for some time, Immigration NZ had been experimenting with a data modelling tool to manage migration, and more specifically, identify ‘undesirable’ immigrants and prioritize their deportation. The project was so secretive that not even New Zealand’s Minister of Immigration was aware that it was ongoing (RNZ Morning Report, 2018). As for the final publication of information on how the New Zealand government uses operational algorithms to manage migration, this came about as a result of experts calling for the government to provide more systematic transparency on if and how it was experimenting and deploying algorithms (Gavaghan et al., 2019). The issue of transparency as a result of procuring technology has not been one which the New Zealand of government has had issues with yet, as the government agencies deploying AI have built their AI systems in-house (RNZ Morning Report, 2019).

Finally, regarding the U.S., information on the use of AI is not easily accessible and much of the available information is through the efforts of advocacy and research groups.  Nevertheless, it is remarkable the type and quantity of data collected, including  real-time information and jail booking, cataloging arrested individuals’ vehicle registration information, insurance claims, credit history, payday loans, public court records, employer records, wire transfers and Taxpayer Identification Numbers, Department of Motor Vehicles records, real property data, professional licenses, criminal and court records, information on individuals’ healthcare providers, their consumer and credit bureau information, incarceration and arrest records, business data, data from social networks, chatrooms and blogs.

### Vulnerability and human rights

The use of these technologies raises a plethora of human rights questions. Immigrants and asylum seekers are a particularly vulnerable group with few easily accessible avenues to contest unfair practices. Immigrants often run into issues understanding the language and navigating the legal system of foreign countries. Additionally, in the case of refugees who are fleeing their home country to find refuge in another, there are often more pressing issues than data protection. It is important to consider, in the case where a refugee is faced with having to decide between pursuing legal action due to a data breach, the decision to be made can be viewed as one between their privacy and their ability to find refuge in the receiving state.

The use of AI to manage migration raises questions around how the migrant is ‘dignified,’ as the UNHCR claims. The UNHCR positions the use of its technology as one which helps the refugee, who otherwise is unidentifiable. However, AI in this case does not only identify the refugee: it makes them completely opaque, where much of their information is available at the click of a button and they are effectively stripped of their privacy. This kind of traceability has led to incidents of self-harm through damaged fingertips (BBC, 2004). Furthermore, through the use of biometric data to access services, migrants must decide between aid or their data privacy (Staton, 2016). The technology also presents challenges for the family unit, as each household is required to select one cash collector (Smit, 2020, p. 18) whose iris is linked to their World Food Programme allowance, who then becomes the only individual who can access the account. Accordingly, “such actions can impact household power dynamics, since women registered as heads of households are given control over the family’s resources (e.g. food and shelter) in communities where traditionally men are the decision-makers” (Schoemaker et al., 2021, p. 14).

## Conclusion

Following the 9/11 attacks, many states and international organizations began to look to innovative technologies to fortify borders (Security Document World, 2016). Alongside these efforts, under the guise of security, states and international organizations have also looked to innovative technologies to efficiently implement their migration or asylum management policies and programs. As evidenced by the cases studied throughout this article, AI has introduced many ways for states and organizations to achieve their immigration objectives. Actors’ intentions, whether pro- or anti-immigration, drive the introduction and use of AI tools to manage migration. The UNHCR uses AI tools, specifically Accenture’s Biometric Matching Engine, to ensure “everyone can exercise the right to seek asylum and find safe refuge in another State, and to return home voluntarily” (UNHCR, 2007). The New Zealand government has introduced AI tools to increase efficiency in “[improving] border security and [making] immigration easier” (Immigration New Zealand, n.d.). Finally, the U.S. uses various AI tools, including a biometric database, biometric and fingerprint matching algorithms, the Investigative Case Management system and data scraping technology to track, locate and ultimately drive the deportation of undocumented migrants.

The aforementioned use cases show that even despite well-intentioned efforts, the decision to use AI as a tool to increase efficiency and support the implementation of migration or asylum management policies and programs often involves jeopardizing or altogether sacrificing individuals’ human rights, including privacy and security, and raises concerns about vulnerability and transparency. Intention certainly plays a central role in the overall use of AI tools to manage migration, however, the technology often requires a decision to trade rights for access, or an eye for an ‘I’.

## Data Availability

The author confirms the data supporting this research are available within the article and cited accordingly in the Bibliography.

## Abbreviations

AI: Artificial intelligence
AWS: Amazon Web Services
BIMS: Biometric Identity Management System
DHS: Department of Homeland Security
EID: Enforcement Integrated Database
ERO: Enforcement and Removal Operations
FALCON-SA: FALCON Search and Analysis
GOST: Giant Oak Search Technology
HART: Homeland Advanced Recognition Technology
HIS: Homeland Security Investigations
ICE: Immigration and Customs Enforcement
ICM: Investigative Case Management
IDENT: Automated Biometric Identification System
IOM: International Organization for Migration
NZ: New Zealand
PRIMES: Population Registration and Identity Management Ecosystem
U.S.: United States
UNHCR: United Nations High Commissioner for Refugees

## Footnotes

1 Immigration NZ only describes the use of operational algorithms in relation to ‘customers.’.
